# Rnalib: a Python library for custom transcriptomics analyses

**DOI:** 10.1093/bioinformatics/btae751

**Published:** 2024-12-24

**Authors:** Niko Popitsch, Stefan L Ameres

**Affiliations:** Max Perutz Labs, Vienna Biocenter Campus (VBC), Vienna A-1030, Austria; Department of Biochemistry and Cell Biology, Max Perutz Labs, University of Vienna, Vienna A-1030, Austria; Max Perutz Labs, Vienna Biocenter Campus (VBC), Vienna A-1030, Austria; Department of Biochemistry and Cell Biology, Max Perutz Labs, University of Vienna, Vienna A-1030, Austria; Institute of Molecular Biotechnology, IMBA, Vienna Biocenter Campus (VBC), Vienna A-1030, Austria

## Abstract

**Motivation:**

The efficient and reproducible analysis of high-throughput sequencing datasets necessitates the development of methodical and robust computational pipelines that integrate established and bespoke bioinformatics analysis tools, often written in high-level programming languages such as Python. Despite the increasing availability of programming libraries for genomics, there is a noticeable lack of tools specifically focused on transcriptomics. Key tasks in this area include the association of gene features (e.g. transcript isoforms, introns or untranslated regions) with relevant subsections of (large) genomics datasets across diverse data formats, as well as efficient querying of these data based on genomic locations and annotation attributes.

**Results:**

To address the needs of transcriptomics data analyses, we developed *rnalib*, a Python library designed for creating custom bioinformatics analysis methods. Built on existing Python libraries like *pysam* and *pyBigWig*, *rnalib* offers random access support, enabling efficient access to relevant subregions of large, genome-wide datasets. *Rnalib* extends the filtering and access capabilities of these libraries and includes additional checks to prevent common errors when integrating genomics datasets. The library is centred on an object-oriented Transcriptome class that provides methods for stepwise annotation of gene features with both, local and remote data sources. The *rnalib* Application Programming Interface cleanly separates immutable genomic locations from associated, mutable data, and offers a wide range of methods for iterating, querying, and exporting collated datasets. *Rnalib* establishes a fast, readable, reproducible, and robust framework for developing novel transcriptomics data analysis tools and methods.

**Availability and implementation:**

Source code, documentation, and tutorials are available at https://github.com/popitsch/rnalib.

## 1 Introduction

The advent of novel biotechnological approaches has led to the generation of complex biological datasets, necessitating the development of efficient, well-tested, and reproducible analysis pipelines. Typically, such pipelines integrate well-established bioinformatics tools with custom scripts developed in high-level programming languages such as Python, R, or Rust. Python, although not always the fastest option in terms of processing speed, is a popular choice among analysts, mainly due to its extensive library availability, its simple and well-documented syntax, and its broad utility in the emerging data science field. Consequently, an increasing number of Python Application Programming Interfaces (APIs) for the handling of genomics data are being developed. Some APIs, such as *pysam* (Heger *et al.* 2009, Danecek *et al.* 2021) and *pybedtools* (Dale *et al.* 2011) were developed as wrappers around well-established and efficient bioinformatics tools like *samtools* (Danecek *et al.* 2021) and *BEDTools* (Quinlan and Hall 2010), respectively. Others, such as *bioframe* (Abdennur *et al.* 2024) or *bionumpy* (Rand *et al.* 2022) are built on top of popular data wrangling and analysis libraries such as *pandas* (The pandas development team 2024) or *numpy* (Harris *et al.* 2020). While these libraries provide efficient access to genomics data sets, they lack direct support for specific use cases typically associated with the development of novel transcriptomics analysis methods. Such use cases include accessing spliced and translated transcript sequences or programmatic annotation, filtering and analysis of gene feature hierarchies.

Here, we introduce *rnalib*, a Python library designed to facilitate the development of novel bioinformatics analysis methods for interpreting transcriptomics data. *Rnalib* implements a Transcriptome class that represents genomic features—such as genes, transcripts, or exons—parsed from a GTF or GFF3 file as Python dataclasses. Hierarchical feature relationships are explicitly modelled, enabling users to navigate them as Python object references. This approach increases code readability and enables efficient programmatic access, including filtering via Python list comprehension.

*Rnalib* provides secure methods for referencing and annotating features by structuring them into immutable genomic locations, which can be used as keys in mapping data structures, and mutable annotations of arbitrary data type. Users can sequentially associate features with genomics data by using a comprehensive set of iterators that offer random access to the most common genomics data formats such as BAM, BED, VCF, and BigWig (see Supplementary Table S1). Random access enables the efficient access to genomic sub regions in such files without the need to sequentially read through the whole dataset.

*Rnalib* iterators provide a uniform interface to genomics data sources and are built on top of existing, efficient Python implementations, like *pysam*, *pyBigWig* (Ryan *et al.* 2023), and *bioframe*. To avoid common pitfalls in genomics data integration, *rnalib* includes several mechanisms to handle deviations in coordinate systems, unsorted data files, contradictory chromosome orders, and mutable genomic interval implementations (see Supplementary page 6ff).

*Rnalib* provides efficient access to and querying of feature sequences and annotations by maintaining respective data structures and access methods. For example, users can access the mature (spliced) RNA sequence of a transcript which is automatically sliced and transformed from the respective gene sequence. They can also directly access genes or transcripts by their IDs and names or perform range queries, which are handled through a combination of interval tree queries and list comprehensions.

**Figure 1.**
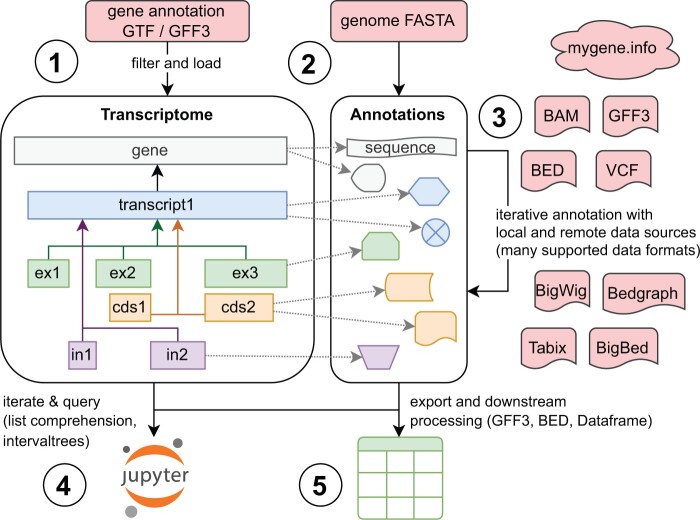
A typical application scenario for *rnalib*: (1) A transcriptome, object is instantiated from a filtered GFF3 gene annotation file. The hierarchical relationships between genes, transcripts, and sub-features (exons, introns, CDS, etc.) are explicitly modelled as Python objects. (2) Sequences for the instantiated gene intervals are loaded from a reference genome FASTA file and stored in an annotation dict. Sequences of sub-features are sliced from there on request. Thereby, *rnalib* avoids storing redundant information (i.e. explicit sub-feature sequences). The same mechanism can also be used for sliceable custom annotations. (3) Transcriptome features are sequentially annotated with genomics data using *rnalib* iterators that support a wide range of common data formats as well as any tabix-indexed data file. Gene annotations can directly be queried from MyGene.info which collates up-to-date annotation data from many large public databases including *Ensembl*, *UniProt*, or *PharmGKB*. (4) Users can conveniently access the data, e.g. via interactive *Jupyter* notebooks. Efficient querying via standard Python list comprehension or (location based) intervaltree queries is supported. (5) Finally, data can be exported in various formats for downstream processing. This includes BED/GFF3 files but also *pandas*DataFrames with customisable columns that can then be further processed, e.g. plotted with *matplotlib*/*seaborn* or analysed with *bioframe*.

Finally, *rnalib* implements various methods to export collated datasets, such as pandas DataFrames, facilitating seamless integration with other genomics libraries and tools. A typical application scenario for *rnalib* is illustrated in Fig. 1.

## 2 Methods

*Genomic intervals*: *Rnalib* references genomic locations using read-only NamedTuples, referred to as genomic intervals (GIs), which can be reliably used as keys in dictionaries and other mapping data structures. Coordinate components of GIs (chromosome, 1-based start and stop coordinates, strand) can be unrestricted, allowing them to reference, for example, entire chromosomes or the first 100k bases of any chromosome. GIs are grouped by chromosomes/contigs of a given reference genome, with their order and lengths being maintained by a reference dictionary (RefDict), an extension of a regular Python dict. RefDicts are typically instantiated automatically from indexing data structures (e.g. ‘.tbi’ or ‘.bai’ files) and are used throughout *rnalib* to ensure compatibility of genomic datasets. *Rnalib* implements a comprehensive set of methods for comparing, sorting, and integrating (sets of) GIs.

*Transcriptome*: *Rnalib* implements a Transcriptome class that facilitates convenient and efficient access, filtering, and annotation of genomic features. Briefly, a (filtered) input GTF/GFF file is parsed and respective (frozen) dataclasses, derived from a generic Feature class (itself a genomic interval), are instantiated. Missing features, such as introns, may be created automatically, and configurable sets of attributes are parsed from the GFF info section and associated with each feature. The implementation tracks parent/child relationships (e.g. between genes, transcripts, and their exons) and enables efficient iteration, querying, and annotation. Data fields, annotations, and parent/child relationships can be accessed via ‘dot’ notation (e.g. ‘<transcript>.gene.gene_name’ to access the gene name associated with a given transcript object). Transcriptome objects can be instantiated from popular gene annotation sets as published by *ENCODE*, *Ensembl*, *UCSC*, *CHESS*, *MirGeneDB*, *WormBase*, and *FlyBase*, but also from user-defined formats (see Supplementary Table S1). Loaded annotation sets can be filtered, for example, by genomic region, feature IDs, or gene types, and gene name aliasing is supported. Transcriptome objects maintain data structures (dictionaries and interval trees) for fast lookup or querying of features and an annotation dictionary for associating arbitrary annotation data with genomic features. Data that annotates all represented nucleotides of a feature may be associated with parent features only and will dynamically be extracted for any descendant (enveloped) feature, minimizing memory usage. For example, nucleotide sequences can be automatically loaded from reference genome FASTA files and are stored in respective gene features only. If the sequence of a child feature (e.g. the intron of a particular transcript) is accessed, the respective subsequence is sliced from the gene sequence (see Supplementary Fig. S1). *Rnalib* also supports access to spliced and translated transcript sequences.

*Iterators*: *Rnalib* implements genomic iterators for efficient, sorted iteration and integration of (parts of) genomics datasets. Supported file formats include *BAM*, *VCF*, *BigWig*, and many more (see Supplementary Table S2). Most iterators are based on third-party libraries, for example, *pysam* or *pyBigWig*, but extend them with additional filtering methods and sanity checks to avoid common errors when integrating multiple genomics datasets. Iterators provide a clean separation between yielded data and referenced genomic location (GI) which facilitates genomic data integration tasks. They can be grouped (by location overlap) or tiled (by tiles of given length) and merged with other iterators. A special AnnotationIterator can be used to associate genomic intervals with overlapping data from multiple other iterators which is useful in many data integration scenarios (see Supplementary Fig. S2). For example, the annotate(.) method of an *rnalib*Transcriptome object uses this iterator to iterate its features and apply a user-provided annotation function to each yielded item (like pandas’ apply(.) method). This method has access to the annotated feature, its Transcriptome object, and all overlapping locations and data from all configured iterators. This enables developers to focus on data integration (e.g. by calculating annotation values from the respective data) without writing extensive boilerplate code or dealing with genomic interval arithmetic and coordinate conventions of the underlying data formats. Additionally, *rnalib* supports the direct annotation of gene objects with remote data from MyGene.info (Xin *et al.* 2016) which collates a large number of public annotation data sets.

*Data export*: Any *rnalib* iterator can be converted into a BED file or a *pandas*DataFrame for further processing by other libraries (e.g. *bedtools* or *bioframe*). Annotated and possibly filtered Transcriptome objects can also be exported as GFF3 files for integration into downstream bioinformatics pipelines.

*Tools and tutorials*: *Rnalib* includes a growing number of Python tools (e.g. for annotating nucleotide conversions or for extracting nucleotide mismatch profiles from BAM files) that implement a command-line interface and can be readily integrated into any bioinformatics pipeline. It also includes several Jupyter notebooks showcasing more complex usage scenarios and interactions with other genomics libraries.

*Performance*: We compared *rnalib* to *bioframe*, a recent genomics library based on *pandas*DataFrames, *HTSeq*, a Python package for analysis of high-throughput sequencing data, and *pybedtools*, a Python library wrapping the popular BEDTools utilities. Each library implements different data access approaches with advantages and disadvantages in different access scenarios:

- *Bioframe* leverages the efficient implementation of *pandas*DataFrames but requires loading whole datasets into memory before they can be processed.
- *HTSeq* is, like *rnalib* based on *pysam*, but is more general and exposes *pysam’*s random access features for only few data formats (e.g. BAM).
- *Pybedtools* is built on the efficient implementation of *BEDTools* but lacks random access features, requiring the preprocessing of entire datasets when just a small subset is needed.
- *Rnalib* is built on the efficient implementation of *pysam* and *pyBigWig* and leverages indexing data structures of genomics datasets, such as *Tabix* (Li 2011), for efficient random access.

The results of these comparisons are provided in the Supplementary (page 8ff). Overall, *rnalib* greatly outperformed existing approaches when focussing on data subsets due to its efficient random access features, while iteration of whole datasets required similar or longer processing time.

## 3 Conclusion

*Rnalib* is a Python library specifically designed for transcriptomics analyses. It enables users to instantiate Transcriptome objects by loading (filtered) gene annotation files from popular providers. Transcriptome objects explicitly model hierarchical relationships between genomic features and *rnalib* supports their efficient, sequential annotation through random access to a wide range of genomics datasets. Access and data integration is facilitated by a clean API that distinguishes between genomic locations and associated data. The resulting annotated and filtered data can be efficiently queried and exported for further analysis by other bioinformatics tools. The library includes a comprehensive test suite based on a rich collection of curated genomic test data and published datasets from other established libraries. The target audience for *rnalib* includes bioinformatics analysts and developers, and its primary design goal is to enable fast, readable, reproducible, and robust development of novel bioinformatics tools and methods.

## Supplementary Material

btae751_Supplementary_Data

## Data Availability

Source code is available at https://github.com/popitsch/rnalib.
