# Supplementary material for: Rnalib: a Python library for custom transcriptomics analyses
Source: Bioinformatics. 2024 Dec 24;41(1):btae751. doi: 10.1093/bioinformatics/btae751 (PMC11734754; doi:10.1093/bioinformatics/btae751)
Supplement: btae751_Supplementary_Data [file btae751_supplementary_data.pdf]

Rnalib: a Python library for custom transcriptomics analyses
Supplement

This document contains additional information and figures describing the *rnalib* Python library.

Contents

Sequence Slicing Example 2

Supported GFF3 flavours 3

Genomic Iterators 4

AnnotationIterator example 5

Common pitfalls when handling genomics data 6

Performance comparison 8

References 13

Sequence Slicing Example

Annotations that reference all represented nucleotides of a feature may be associated with parent features only and will then dynamically be sliced from there for any descendant (enveloped) feature which minimises required memory, however, at the cost of traversing the feature hierarchy and one slicing operation per request (here: 2x2 steps from exon to gene and 2 slicing operations for the two exons).

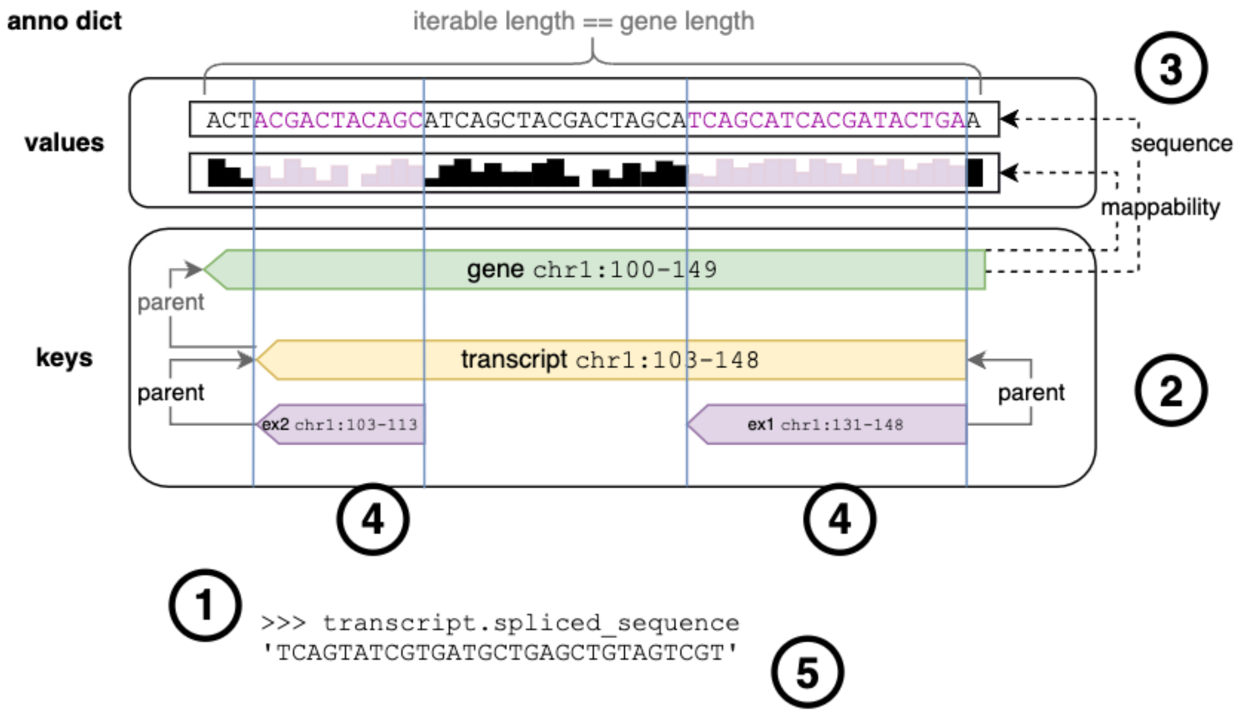

Figure S1: Sequence slicing example. The sketch shows the annotation dict of a *rnalib* transcriptome with keys (transcriptome features) in the bottom box and associated annotations on top of it. The dict contains four features annotated on the reverse strand that are linked by parent/child relationships: a gene, a transcript and two exons. Only the gene feature is associated with two different annotation values (slicable iterables), one containing the respective nucleotide *sequence* (as read from a FASTA file) and one containing a *numpy* array of same length containing *mappability* values (depicted as bargraph). If a user now accesses the spliced sequence of the transcript (1), then *rnalib* first retrieves the sequence of both contained exons by traversing the feature hierarchy (2) until it finds the respective sequence annotation associated with the gene feature (3) and then slices the respective subsequence from there based on the relative coordinates of the (enveloped) child features (4). The exon sequences (depicted in magenta colour) are then reverse complemented (as the transcript is annotated on the reverse strand), concatenated and returned (5). Accessing the *mappability* values of individual exons would work accordingly (without the reverse complement and concatenation operations).

Supported GFF3 flavours

Provider	Data format	Reference
Gencode	GFF3, GTF	https://www.gencodegenes.org/
Ensembl	GFF3, GTF	https://www.ensembl.org/
Chess	GFF3, GTF	https://ccb.jhu.edu/chess/
UCSC	GTF	https://genome.ucsc.edu/
MirGeneDB	GFF3	https://mirgenedb.org/
Flybase	GTF	https://flybase.org/
Wormbase	GFF3	https://wormbase.org/
Generic	GFF3, GTF	NA

Table S1: List of currently supported GFF3 flavours. Users can directly instantiate transcriptome objects from the respective annotation files. Please note that this list will likely be extended in the future depending on user feedback and author contributions.

Genomic Iterators

**LocationIterator**	**Iterated data format/structure**	**Yielded data (type)**
MemoryIterator	Dicts and iterables	Arbitrary data
TranscriptomeIterator	Transcriptome features	Feature annotations
FastaIterator	FASTA	Nucleotide characters
TabixIterator	Tabix-indexed TSV files	Tuple
BedGraphIterator	Bedgraph	Float
BedIterator	BED	BedRecord (BED12)
BigBedIterator	BigBED	BigBedRecord
BigWigIterator	BigWIG	Float
VcfIterator	VCF	VcfRecord
GFF3Iterator	GFF3, GTF	Dict of annotation values
PandasIterator	Pandas DataFrame	Tuple
BioframeIterator	Bioframe Pandas DataFrame	Tuple
PyrangesIterator	Pyranges Pandas DataFrame	Tuple
PybedtoolsIterator	PyBedTools BedTool	PyBedTools intervals
ReadIterator	BAM	Pysam AlignedSegment, tuple of mismatches (optional)
PairedReadIterator	BAM (paired end)	ReadPair (tuple of pysam AlignedSegments and mismatch tuples)
FastPileupIterator	BAM	Counter of Counters
GroupedLocationIterator	LocationIterator	Tuple of grouped locations and data
TiledIterator	LocationIterator	Tuple of tile data
MergedLocationIterator	LocationIterators	Tuple of data and labels
AnnotationIterator	LocationIterators	Tuple of data and locations

Table S2: Implemented location iterators, supported data formats/structures and yielded data items. Note that *rnalib* also implements a few iterators (e.g., a FastqIterator) that are not location iterators and yield only data items (e.g., reads).

AnnotationIterator example

Rnalib contains a special `AnnotationIterator` for annotating genomic intervals with data from one or multiple other genomic location iterators. This iterator synchronizes genomic locations of the primary iterator (iterating the intervals to be annotated) with all annotating iterators and yields respective locations and data for all overlapping intervals. Figure S2 illustrates one primary iterator (blue intervals) that is annotated with three others (green, black and magenta intervals). The box on the bottom shows the expected results (top to bottom): the blue intervals are iterated and all overlapping intervals from the other iterators are reported including their locations and data. A respective code example is included in the *rnalib* tutorials.

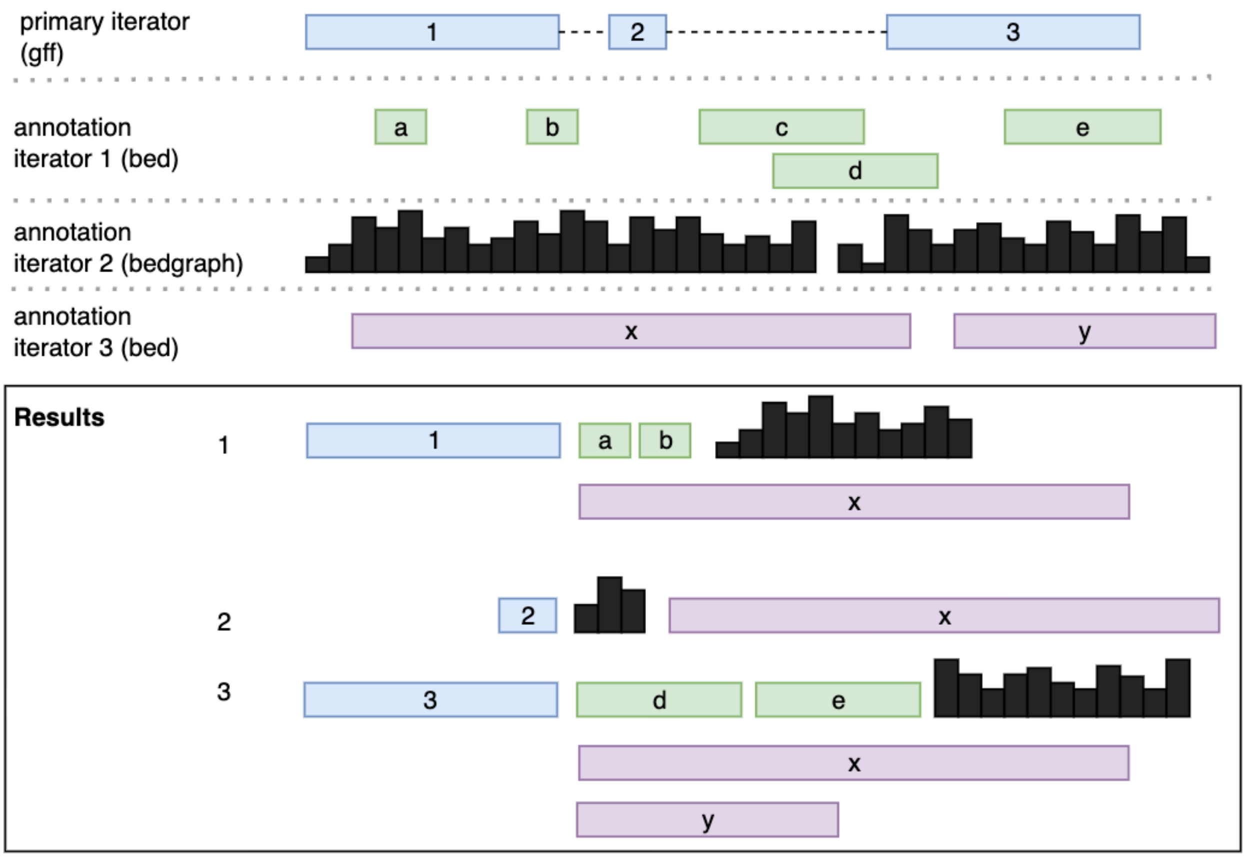

Figure S2: `AnnotationIterator` example. Here, a primary GFF iterator (blue) is annotated with data yielded from three iterators (green, black and magenta). The annotation iterator yields all primary intervals, associated with all overlapping intervals from all annotation iterators. Users have immediate access to all data and all respective genomic intervals but do not need to care about genomic interval arithmetic or coordinate conventions of the underlying data formats. This helps them to fully concentrate on the respective data integration tasks.

Common pitfalls when handling genomics data

In this section we describe some common pitfalls that regularly hamper bioinformatics analyses leading to wrong results:

- unsorted input files
- wrongly interpreted coordinate systems
- mutable interval implementations

We showcase those pitfalls using realistic scenarios and discuss how we addressed these issues in *rnalib*. Notably, in most cases analyses run through and deliver plausible (yet wrong) results which makes these kinds of errors hard to catch.

Unsorted input files. Genomic data files that are not properly sorted by genomic coordinates are a common error source when accessing genomic data files. Most tools and algorithms assume their input files to be sorted and often do not even implement respective checks which might lead to wrong results that are hard to identify. As an example, we show how unsorted input may lead to wrong analysis results using the *pybedtools* library (Dale, et al., 2011).

The following code block (Figure S3) was copy-pasted from the *pybedtools* page (Date: May 2024), and the idea of this example is to create a list of gene names that are <5 kb away from intergenic SNPs. However, this code does not work properly (with *pybedtools* v0.9.1) due to inconsistent chromosome order of the two input files (hg19.gff: chr1, chr21; snps.bed.gz: chr21, chr1; both being *pybedtools* test resources). As a result, the `genes.closest()` method reports only -1 as distance which is why all closest genes will be reported, not just the ones at max 5kb distance. Notably, the code runs without errors/warnings and returns a reasonable-sized list of gene names which makes it hard to spot the error. Only when omitting the `stream=True` flag, `genes.closest()` fails and reports the inconsistent chromosome order.
Note, that in *rnalib*, input files always must be *bgzipped* and indexed which requires them to be coordinate sorted and that `RefDict`'s of different data files are compared to ensure consistent chromosome ordering.

```python
def run_pybedtools_example(gff_file, snp_file):
    snps = pybedtools.BedTool(snp_file)
    genes = pybedtools.BedTool(gff_file)
    intergenic_snps = snps.subtract(genes).saveas()
    nearby = genes.closest(intergenic_snps, d=True, stream=True).saveas()
    nbgenes = [gene.name for gene in nearby if int(gene[-1]) < 5000]
    display(f"We found {len(nbgenes)} nearby genes. Unique names: {len(set(nbgenes))}")


display("Unsorted input files:")
run_pybedtools_example(rna.get_resource("pybedtools::hg19.gff"), rna.get_resource("pybedtools::snps.bed.gz"))
display("Sorted input files:")
run_pybedtools_example(rna.get_resource("pybedtools_gff"), rna.get_resource("pybedtools_snps"))
```

```
'Unsorted input files:'
'We found 4217 nearby genes. Unique names: 4215'
'Sorted input files:'
'We found 2422 nearby genes. Unique names: 2419'
```

Figure S3: Different results when applying pybedtools to unsorted and sorted data files.

Wrongly interpreted coordinate systems. Another common pitfall when integrating genomics datasets is that genomics data formats differ in their representation of coordinates and intervals. Respective coordinate systems can be 0 or 1-based and intervals can, e.g., be half-open or closed. These differences need to be considered when combining (integrating) respective datasets which is not always done automatically by common tools/libraries.

In *bioframe (Abdennur, et al., 2024)*, for example, interval coordinates are "... assumed to be 0-based and intervals half-open (1-based ends) i.e. (start, end)" as, for example, in the BED file format. However, this is not enforced or validated when reading genomic datasets in different formats (e.g., GFF which is 1-based) which results in *bioframe* wrongly interpreting 1-based as 0-based coordinates. Consequently, start coordinates of misinterpreted datasets are shifted by 1bp which may lead to subtle errors in downstream analyses. A respective example is included in the *rnalib* tutorials.

Note, that *rnalib* provides format-specific iterators that ensure proper coordinate interpretation which makes it harder (yet not impossible) to fall into this pit.

Mutable interval implementations. Another, more subtle, pitfall results from how genomic intervals and their annotations are implemented in the different Python libraries. In most cases, intervals are implemented as *mutable* objects whose values can be changed at runtime. Often these interval objects also contain associated (mutable) meta-data (such as interval names, scores, etc.). This, however, means that updates of intervals or associated meta-data results in changed object IDs and hash values which makes them unsafe to use as keys in mapping data structures such as `dicts` and can lead to unwanted side-effects. A respective example is included in the *rnalib* tutorial.

In *rnalib*, genomic intervals and their annotations are modelled separately with the former being implemented as named tuples or frozen (basically immutable) `dataclasses` while the latter being stored in a dict-like data structure that is indexed by the respective genomic interval.

Performance comparison

In this section we informally compare *rnalib*'s iteration speed with different genomics libraries in different scenarios and plot the results. Note that many factors are influencing these benchmarks (such as I/O speed, memory consumption, file composition, etc.) so be careful when drawing conclusions. All present tests were executed on a MacBook Pro M3 Max with 64GB RAM. While the presented numbers do not represent a systematic, formal performance evaluation, they should still be indicative of the relative speed of the different approaches in various application scenarios.
We compared *rnalib* to *pybedtools (Dale, et al., 2011)*, a Python library wrapping the popular *BEDTools* utilities, *bioframe (Abdennur, et al., 2024)*, a genomics library based on *pandas* DataFrames (The pandas development team, 2024) and *HTSeq (Anders, et al., 2015)*, a Python package for analysis of high-throughput sequencing data.

Calculate average feature lengths. In this first test, we compare different ways to calculate the average feature length in a medium-sized (800k intervals) bed file (*snps.bed.gz* from the *pybedtools* testdata repository). Figure S4 plots million iterations per seconds for the different methods we compared:

- *rnalib*'s `BedIterator` is faster than *pybedtools* which relies on the BEDtools implementation for iterating the full bed file.
- The *bioframe* approach is much faster for this task as it first loads the whole dataset into a *pandas* `DataFrame` that then allows efficient calculation of mean feature lengths.
- The *HTSeq* approach is fastest as it directly reads and parses the BED file without additional overhead.

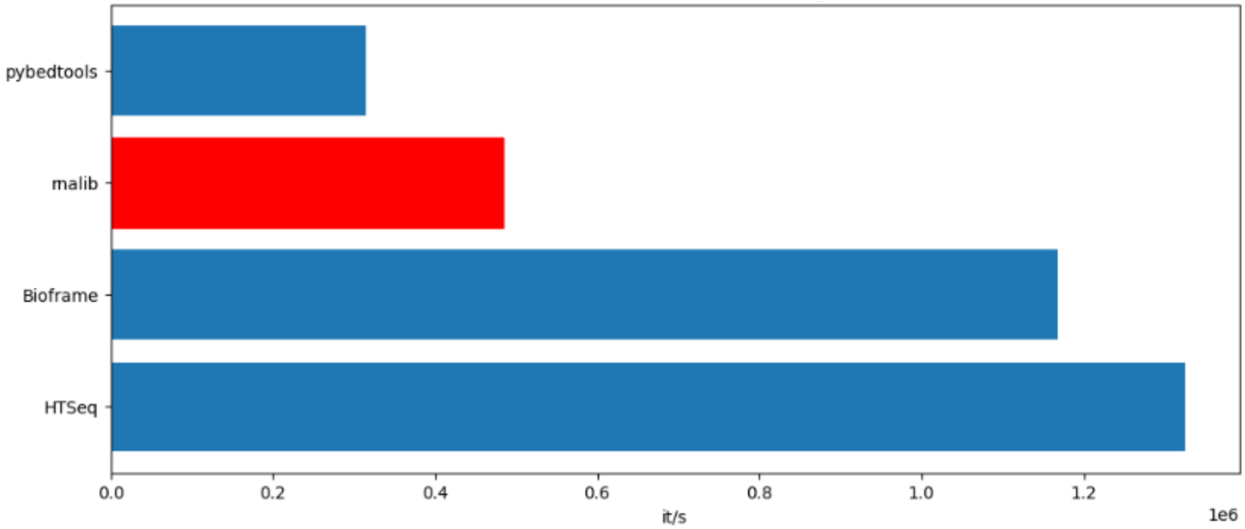

Figure S4: Performance comparison for calculating average feature size in a BED file with 800k intervals.

Value-filtered iteration. Here we compared various methods for iterating all intervals in a BED file and filtering for entries with a given minimum length. We compared *HTSeq*'s `BED_Reader`, *bioframe* and *pybedtools* iterators with various filter options and *rnalib*'s `BedIterator` (Figure S5).

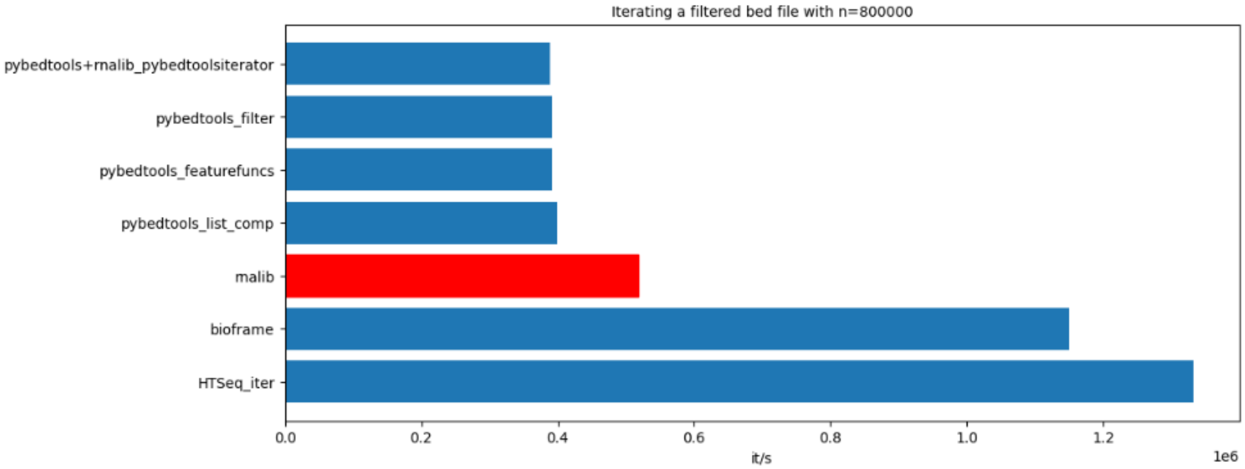

Figure S5: Performance comparison for value-filtered iteration. *Pybedtools_filter*: *pybedtools* with filter command using lambda function; *Pybedtools_featurefuncs*: *pybedtools* with filter command using pybedtools featurefuncs command; *Pybedtools_list_comp: pybedtools with filtering via Python list comprehension;* pybedtools+rnalib_pybedtoolsiterator: as *Pybedtools_featurefuncs but file is then saved and iterated via rnalib.*

In this scenario, no pre-filtering based on genomic location is possible and *HTSeq* is the fastest option due to its minimal overhead when iterating the full BED file, closely followed by *bioframe*. *Rnalib* finishes third, faster than the compared *pybedtools* approaches.

Grouped aggregation. Here we tested grouped aggregation of some property using random datasets. First, we created a data frame with random intervals (n=1e4,1e5,1e6,1e7) and random values between [0; 1000] and stored them as bgzipped+tabixed *bedgraph* files. Then we tested different methods for calculating a chromosome-grouped mean score (Figure S6).

- For *pybedtools*, we intersected the file per chromosome, stored the values in a *numpy* array (Harris, et al., 2020) and then calculated the mean.
- In a second *pybedtools* approach, we iterated the whole file and summed and counted values in a per-chromosome dict. We then calculated the mean from these values.
- We used a corresponding approach for the *HTSeq* `BED_Reader`.
- For *bioframe*, we used the *rnalib* `BioframeIterator` class just for loading the dataframe and then grouped/aggregated directly with *pandas* functionality.
- We use *rnalib*'s `BedGraphIterator` for iterating the values per chromosome, stored them in a *numpy* array and calculated the mean.

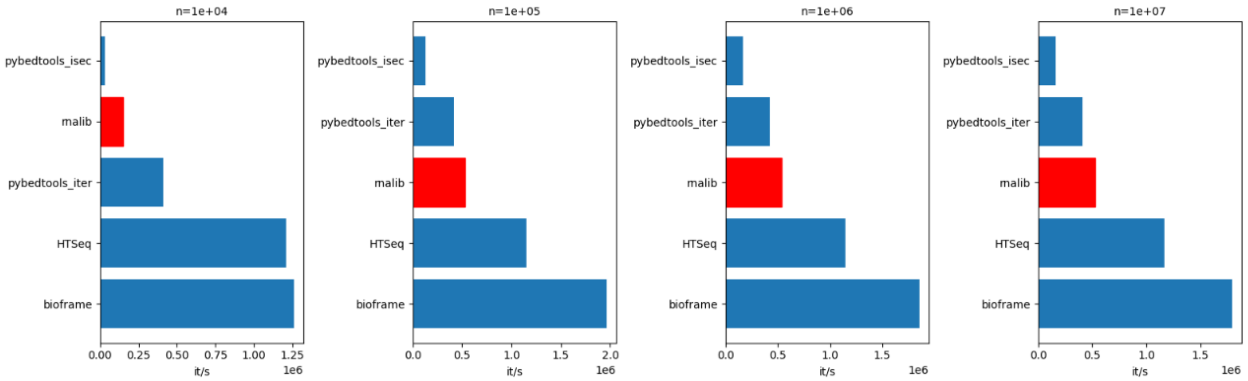

Figure S6: Performance comparison for calculating grouped scores for different methods. *Pybedtools_isec*: *pybedtools* with intersection; *Pybedtools_iter*: *pybedtools* whole dataset iteration, see text.

In a summary,

- *Bioframe* was the fastest option for this task due to the efficient *pandas* data grouping/aggregation functionality.
- *HTSeq* performance was close to *bioframe* for small datasets but clearly slower when larger files were considered.
- *rnalib*'s `BedGraphIterator` finished third as per-chromosome grouping is more efficiently handled than intersections in pybedtools.
- *Pybedtools* iteration is slower than *rnalib and pybedtools* intersection per chromosome is the slowest approach in this scenario.

Location-filtered iteration. Here we finally add additional filtering steps and compare methods for counting minus strand features on one chromosome in a large GFF3 file that was downloaded from *GENCODE* (https://www.gencodegenes.org/), sorted, compressed, index and analysed. We restricted this analysis to human chromosome 21 only (Figure S7).

- for *pybedtools*, we either intersected the file with the respective region of interest (pybedtools_intersect) or applied a filter method (pybedtools_filter), then parsed the respective fields and counted.
- for *bioframe*, we initialize a chromosome-filtered *pandas* DataFrame via our `BioframeIterator` implementation, filtered for the proper strand and region using pandas and then iterated and counted.
- for *HTSeq*, we iterated over a `GFF_Reader`, filtered by interval strand and counted the returned feature_type.
- for *rnalib*, we used a chromosome filtered `GFF3Iterator` for comparison, filtered by interval strand and counted the returned *feature_type*.

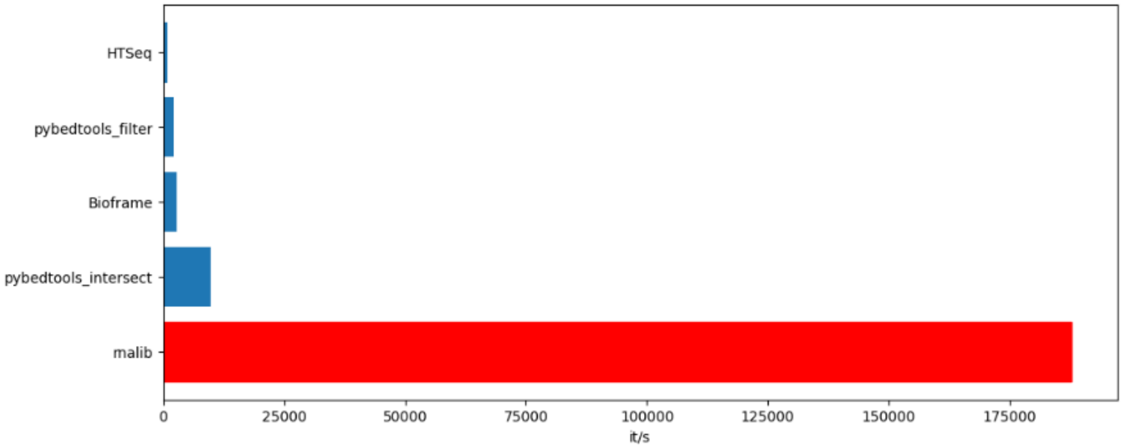

Figure S7: Performance comparison of location-filtered data. *Pybedtools_intersect*: *pybedtools* with intersection; *Pybedtools_iter*: *pybedtools* whole dataset iteration, see text.

In a summary,

- The *HTSeq* approach is slowest. Pre-filtering on chromosome is not possible as no random-access interface is available.
- The *pybedtools_filter* option is also relatively slow as it also needs to iterate the whole dataset when filtering.
- The shown *pandas/bioframe* approach first needs to read the whole `DataFrame` into memory before it can efficiently filter in two steps for the targeted genomic region and strand.
- The *pybedtools_intersect* option is faster as it only needs to iterate the specified chromosome after (the costly) intersection operation.
- Finally, the *rnalib* GFF3Iterator (based on pysam) is by far the fastest option due to the fast chromosome filtering based on the leveraged tabix index.

Pileup performance. Finally, we also compared *rnalib*'s `FastPileupIterator` implementation to *pysam*'s (Heger, et al., 2009) *pileup* method (Figure S8). Our method was ~24X faster and required a much shorter code block that is arguably easier to read/maintain (see online tutorial for code). The `FastPileupIterator`, however, doesn't implement all functionality of *pysam*'s method (for example, it does not report on inserted sequences) and might therefore not be applicable in all usage scenarios. For the present test, we iterated 52,652 positions from a small BAM file.

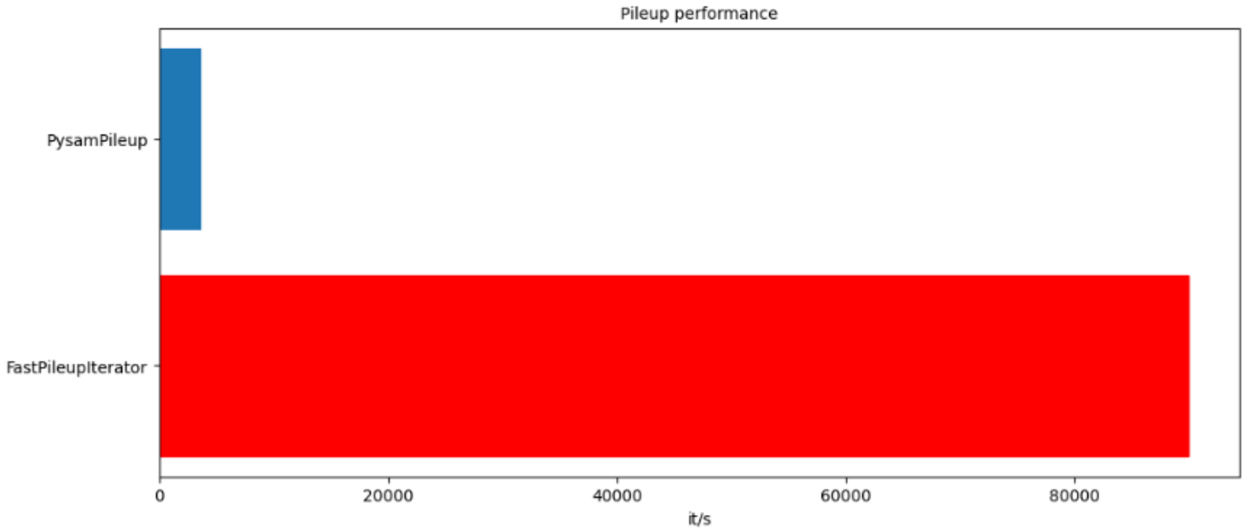

Figure S8: Performance comparison of *pysam's* pileup command and *rnalib's* `FastPileupIterator` based on the iteration of >50k individual alignment positions.

Summary. While *rnalib* is slower than some of the compared approaches when the whole dataset is considered, it greatly outperforms them when only a subregion of the dataset needs to be iterated due to its efficient random-access features that are built on *pysam*'s and pyBigWig's functionality. Please note, that iteration performance was no primary design goal of *rnalib*. This performance comparison should solely demonstrate where our implementation stands with respect to other libraries and highlight its benefits when random access is required.

The source code of all performance comparisons is provided in the 'RelatedWork_performance' notebook in the *rnalib* GitHub repository.

References

Abdennur, N., *et al.* Bioframe: operations on genomic intervals in Pandas dataframes. *Bioinformatics* 2024;40(2).

Anders, S., Pyl, P. and Huber, W. HTSeq—a Python framework to work with high-throughput sequencing data. *Bioinformatics* 2015;31(2):166-169.

Dale, R.K., Pedersen, B.S. and Quinlan, A.R. Pybedtools: a flexible Python library for manipulating genomic datasets and annotations. *Bioinformatics* 2011;27(24):3423-3424.

Harris, C.R., *et al.* Array programming with NumPy. *Nature* 2020;585:357-362.

Heger, A., *et al.* Pysam: HTSlib interface for Python. In.: https://github.com/pysam-developers/pysam; 2009.

The pandas development team. pandas-dev/pandas: Pandas. In.: Zenodo; https://doi.org/10.5281/zenodo.3509134; 2024.

Xin, J., *et al.* High-performance web services for querying gene and variant annotation. *Genome Biology* 2016;17(1).
